# Is There a Relationship between COVID-19 and Hyponatremia?

**DOI:** 10.3390/medicina57010055

**Published:** 2021-01-09

**Authors:** Gina Gheorghe, Madalina Ilie, Simona Bungau, Anca Mihaela Pantea Stoian, Nicolae Bacalbasa, Camelia Cristina Diaconu

**Affiliations:** 1 Department of Gastroenterology, “Carol Davila” University of Medicine and Pharmacy, 050474 Bucharest, Romania; gheorghe_gina2000@yahoo.com (G.G.); drmadalina@gmail.com (M.I.); 2 Department of Gastroenterology, Clinical Emergency Hospital of Bucharest, 105402 Bucharest, Romania; 3 Department of Pharmacy, Faculty of Medicine and Pharmacy, University of Oradea, 410028 Oradea, Romania; simonabungau@gmail.com; 4 Department of Diabetes, Nutrition and Metabolic Diseases, “Carol Davila” University of Medicine and Pharmacy, 020475 Bucharest, Romania; ancastoian@yahoo.com; 5 Department of Visceral Surgery, Center of Excellence in Translational Medicine, Fundeni Clinical Institute, 022328 Bucharest, Romania; nicolae_bacalbasa@yahoo.ro; 6 Department of Internal Medicine, “Carol Davila” University of Medicine and Pharmacy, 050474 Bucharest, Romania; 7 Department of Internal Medicine, Clinical Emergency Hospital of Bucharest, 105402 Bucharest, Romania

**Keywords:** SARS-CoV-2, COVID-19, SIADH, dyselectrolytemia, hyponatremia

## Abstract

Nowadays, humanity faces one of the most serious health crises, the severe acute respiratory syndrome coronavirus 2 (SARS-CoV-2) pandemic. The severity of coronavirus disease 2019 (COVID-19) pandemic is related to the high rate of interhuman transmission of the virus, variability of clinical presentation, and the absence of specific therapeutic methods. COVID-19 can manifest with non-specific symptoms and signs, especially among the elderly. In some cases, the clinical manifestations of hyponatremia may be the first to appear. The pathophysiological mechanisms of hyponatremia among patients with COVID-19 are diverse, including syndrome of inappropriate antidiuretic hormone secretion (SIADH), digestive loss of sodium ions, reduced sodium ion intake or use of diuretic therapy. Hyponatremia may also be considered a negative prognostic factor in patients diagnosed with COVID-19. We need further studies to evaluate the etiology and therapeutic management of hyponatremia in patients with COVID-19.

## 1. Introduction

The severe acute respiratory syndrome coronavirus 2 (SARS-CoV-2) is part of the beta-coronavirus family and causes coronavirus disease 2019 (COVID-19) [1,2]. In March 2020, the World Health Organization (WHO) declared COVID-19 a pandemic, one of the most severe pandemics that humanity has faced over time [1,2]. Worldwide, there have been 45,921,698 cases of SARS-CoV-2 infection and 1,193,909 deaths, these numbers increasing alarmingly with each passing day [3].

In most cases, this viral infection manifests with pneumonia, characterized by fever, dyspnea, cough, and bilateral interstitial infiltrates on chest X-ray examination [4]. According to one study from the USA, among the most frequent symptoms encountered in patients with COVID-19 are cough, fever, myalgia, headache, dyspnea, sore throat, diarrhea, nausea/vomiting, loss of smell or taste, abdominal pain, and rhinorrhea [5]. These patients can also present thrombotic manifestations, conjunctivitis, dermatological findings like maculopapular urticaria, vesicular eruptions, or transient livedo reticularis [6,7]. Some patients may even develop severe clinical forms, with acute respiratory distress syndrome (ARDS), respiratory failure and multiple organ dysfunction and death [8]. In contrast, an important part of the COVID-19 patients can remain asymptomatic, which makes the diagnosis more difficult [4].

## 2. COVID-19 and Hyponatremia

The clinical evolution of patients with COVID-19 can be unpredictable, as these patients can develop a series of complications, as it is summarized in Table 1) [4,9].

## 3. Incidence of Hyponatremia in Patients with COVID-19

Sometimes, patients with COVID-19 can present dyselectrolytemia, like hyponatremia, which is defined by serum sodium levels less than 135 mmol/L. In many cases, this electrolytic disorder is caused by a variety of factors [10,11]. Hyponatremia is the most common electrolyte disorder seen in clinical practice and is associated with increased risk of death [12]. This electrolytic disequilibrium is classified in hypovolemic, euvolemic, and hypervolemic hyponatremia, each category’s therapeutic approach being different [2].

The most recognizable cause of hyponatremia is the syndrome of inappropriate antidiuretic hormone secretion (SIADH), found in about 40–50% of patients with this electrolyte disorder [13]. These percentages may be higher in some conditions, such as traumatic brain injury, subarachnoid haemorrhage or pneumonia [14]. SIADH can occur in the evolution of inflammatory diseases of infectious or non-infectious causes, malignant diseases, cardiovascular or hepatic diseases, but also in the evolution of acute respiratory distress syndrome (ARDS) [14,15]. Ho and colleagues reported the first case of COVID-19 associated with SIADH manifested by new-onset seizures [16]. Yousaf et al. published a series of cases of patients diagnosed with COVID-19 who also associated SIADH [17]. All patients included in this study had severe acute hyponatremia. After excluding other possible etiologies, these authors established that this hydro electrolytic disorder is secondary to SIADH [17].

## 4. Pathophysiology of Hyponatremia in Patients with COVID-19

In infectious diseases (i.e. COVID-19), haemodynamic disorders or an inadequate immune response may cause kidney damage [18]. It is also possible that kidney cells are directly affected by the infection, according to some studies that have demonstrated the presence of viral particles in the proximal tubules and podocytes [18]. Renal cells express receptors and enzymes used by viruses as gateways, such as angiotensin-converting enzyme 2 (ACE2) [9,18]. This enzyme is also found in the lungs, heart and intestines, which explains the damage of these organs in COVID-19 [18]. Another pathophysiological mechanism that may explain the renal impairment in COVID-19 is inflammatory cytokine-induced impairment. It is known that the cytokine cascade can cause a number of renal pathological changes, as well, such as acute kidney injury (AKI), tubular necrosis, dysfunction of the kidney proximal tubule, glomerulopathy and electrolyte abnormalities [9,18,19,20].

Hyponatremia was identified in approximately 35% of patients with pneumonia [2]. The presence of hyponatremia in patients with pneumonia has been associated with a higher mortality rate, indicating the need for an early diagnosis and proper therapeutic management, to improve the prognosis of these patients [2]. The literature also reports that approximately 60% of patients with COVID-19 and watery diarrhea have moderate hyponatremia. In this situation, hyponatremia is possibly secondary to viral replication in the intestinal epithelial cells [21]. Ata et al. report the case of a young patient, known with diabetes mellitus, who presented with diarrhea and abdominal pain. Laboratory investigations identified hyponatremia (120 mmol/L), and the patient subsequently tested positive for SARS-CoV-2 infection. In this case, the cause of hyponatremia was not clear, but the authors suspected the association between SIADH and stool sodium loss to be the culprit [22].

**Figure 1:**
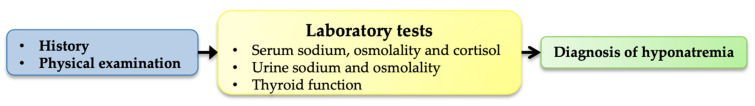
Diagnostic algorithm in hyponatremia.

In order to establish the diagnosis and etiology of hyponatremia, a careful history and physical examination are required; investigations such as serum sodium level, urine sodium level, serum osmolality, urine osmolality, thyroid function tests and serum cortisol may be needed (Figure 1) [23].

In the pathogenesis of hyponatremia, interleukin-6 (IL-6) released by monocytes and macrophages plays an important role; it induces the non-osmotic release of vasopressin and secondary electrolyte disturbances [14]. IL-6 has also been shown to be involved in the pathophysiology of COVID-19 [13,17,24]. This explains the use and efficacy of tocilizumab, a humanized monoclonal antibody against the IL-6 receptor, in the treatment of patients with COVID-19 [25]. Other cause that may lead to increased ADH secretion among patients with SARS-CoV-2 infection is volume depletion through digestive loss (diarrhea or vomiting) or secondary to reduced oral fluid intake [26]. All these lead to water retention and the secondary increase of ADH secretion. Numerous SARS-CoV-2-induced comorbidities, such as pneumonia, respiratory failure, stroke etc., can also contribute to the development of SIADH [26]. The most common cause of death in patients with COVID-19 is ARDS; secondary, the inflammatory syndrome characterized by the massive release of cytokines and multiple organ failure may also contribute to the fatal evolution of these patients [13].

A retrospective study in Italy looked at the clinical impact of hyponatremia and the correlation with IL-6 levels in a group of patients diagnosed with COVID-19 [13]. Out of 29 patients, 15 had low serum sodium levels upon admission. There was an inversely proportional relationship between serum sodium level and IL-6 level and a directly proportional relationship between serum sodium level and PaO_2_/FiO_2_ ratio [14]. The relationship between serum IL-6 and sodium was also suggested by a significant increase in serum sodium 48 h after initiation of tocilizumab treatment [13]. Patients with hyponatremia and COVID-19 have a worse prognosis compared with patients without electrolyte disturbances [13].

A study that included 1099 patients hospitalized for COVID-19 in China showed an average serum sodium value of 138 mmol/L (range 135–141 mmol/L) in these patients [27]. Another study conducted in New York, that involved 5700 hospitalized patients with COVID-19, including 55 solid organ transplant patients, showed an average serum sodium level of 136 mmol/L (133–138 mmol/L) [28].

An article presents the case of a 55-year-old woman with kidney transplant, diagnosed with COVID-19, who associated hyponatremia since hospitalization (serum sodium concentration upon admission <120mmol/L) [29]. This woman reported close contact with a confirmed patient with SARS-CoV-2 infection; she had also suggestive symptoms, respectively low-grade fever at home (temperature 37.4 °C), cough, dyspnea, headache, decreased appetite, nausea, and fatigue [29]. Under these conditions, there has been a suspicion of SARS-CoV-2 infection from the beginning. Another article presents the case of a 57-year-old man with a history of hypertension and type 2 diabetes who presented for dizziness, physical asthenia, headache, and nausea. This patient did not report close contact with a confirmed case; the electrocardiogram and chest X-ray were normal. On admission, however, laboratory tests showed severe hyponatremia (serum sodium level upon admission 112 mmol/L) and the RT-PCR test for SARS-CoV-2 proved to be positive [30]. In all cases of COVID-19 and hyponatremia reported until now, the patients presented with fever and radiological findings consistent with pneumonia [17].

Another study conducted in Hubei, China, evaluated the particularities of patients diagnosed with COVID-19 and disorders of sodium balance [31]. Thus, 1254 patients diagnosed with COVID-19 were enrolled. Of these, 9.9% (124 patients) associated hyponatremia (serum sodium below 135 mmol/L) and 2.4% associated hypernatremia (sodium over 145 mmol/L) [31]. The presence of hyponatremia among these patients was associated with old age, the existence of several comorbidities and diagnosis of severe pneumonia on chest X-ray. Regarding the etiology of hyponatremia, digestive sodium losses, by diarrhea or vomiting, could explain this hydro-electrolytic disorder only in a small number of patients (8.7% diarrhea, 3.3% vomiting) [31]. However, another reason that contributed largely to the development of hyponatremia in patients included in this study was renal failure, with elevated blood urea nitrogen and creatinine levels. A possible explanation could be the advanced age of patients with COVID-19 and hyponatremia and implicitly multiple comorbidities, with impaired renal function. On the other hand, liver function and serum albumin levels were normal in these patients, which significantly reduced the likelihood of hypervolemic hyponatremia of nephrotic or hepatic etiology. Another hypothesis is hyponatremia secondary to cardiac dysfunction. The pathophysiological mechanism incriminated in this situation is the expression by myocytes of angiotensin-converting enzyme 2 (ACE2), which acts as a viral receptor for SARS-CoV-2 and, implicitly, cardiac disease [31]. This study also provides additional evidence for the association of hyponatremia in COVID-19 patients with SIADH. The possible causes of SIADH in this situation include both the positive pressure ventilation (with non-osmotic stimulation of ADH secretion) and the usage of antibiotics and corticosteroids [31]. Regarding symptomatology, hyponatremia in SARS-CoV-2 positive patients was associated with a greater predisposition to fever and nausea, and in terms of biological changes, with increased leukocytes, neutrophils and high sensitivity C-reactive protein (HS-CRP). Hypernatremia was rarer in patients with COVID-19 compared with hyponatremia (2.4% vs. 9.9%) [31]. The only differences between patients with hypernatremia and those with normo-natremia were the clinical complications and biological anomalies [25]. In terms of treatment, patients with hypernatremia were treated more frequently with traditional Chinese medicine, as opposed to those with hyponatremia, who required intensive oxygen supply, high doses of antibiotics and corticosteroids [31]. Related to the duration of hospitalization, no significant differences were identified between patients with hypernatremia and those with normo-natremia, in contrast to patients with hyponatremia, who generally had more severe forms of the disease and required longer hospitalization [31].

In a study on 323 patients with COVID-19, Carvalho et al. concluded that hyponatremia may be a factor pointing towards a bad prognosis. The authors showed that patients with COVID-19 and hyponatremia had higher rates of admission to hospital, intensive care unit transfer, use of artificial ventilation and death, comparatively to patients with COVID-19 and normo-natremia (34% vs. 14%) [32].

Table 2 summarizes the existing studies offering information about the incidence of hyponatremia in COVID-19 patients.

Until now, there are no studies published to show if there is a correlation between the incidence of hyponatremia and the severity of COVID-19, there are only data from case reports. This is an issue that should be clarified by future studies.

## 5. Hyponatremia Management in COVID-19 Patients

There are currently no clinical guidelines for the management of hyponatremia in patients diagnosed with COVID-19. The therapeutic approach of this hydro-electrolytic disorder depends, on one hand, on the etiology, and on the other hand on the volume status and comorbidities of the patient. As specified above, the hyponatremia etiology in patients with COVID-19 is multifactorial. According to existing data, the causes of hyponatremia include SIADH and digestive losses of sodium through diarrhea or vomiting [2]. The pathophysiological judgment is very important, because there are two therapeutic strategies, fluid restriction therapy and electrolytic substitution therapy. Thus, in the case of hypovolemic hyponatremia secondary to gastrointestinal fluid losses, reduced fluid intake or the use of diuretic therapy, guidelines impose initiation of electrolyte replacement therapy [2]. In the case of hyponatremia secondary to SIADH, fluid restriction is needed, that may associate hypertonic saline administration, depending on the level of neurological impairment. This therapeutic approach is relevant to avoid iatrogenic complications, such as pulmonary edema and lung damage exacerbation secondary to SARS-COV-2 infection [2].

## 6. Conclusions

Hyponatremia is frequent among patients with COVID-19, who sometimes may present only with symptoms and clinical signs secondary to this electrolytic imbalance. The diagnosis of hyponatremia upon admission of a patient, in the context of COVID-19 pandemic, should nowadays rise the suspicion of a possible SARS-CoV-2 infection. The causes of hyponatremia in these patients are diverse. It is very important to establish the exact etiology of this electrolytic disorder, because therapeutic management differs depending on its pathophysiological mechanism. Noteworthy, hyponatremia may be considered an unfavorable prognostic factor among patients with COVID-19. Future studies are needed to evaluate the exact incidence, pathogenesis, and therapeutic management of hyponatremia in patients with SARS-CoV-2 infection.

## Figures and Tables

**Table 1:** Complications of patients with COVID 19 [2,5]

Complications	Syndromes, Diseases, Manifestations
**Respiratory**	Acute respiratory distress syndrome (ARDS) Pulmonary embolism
**Cardiac**	Arrhythmias Acute cardiac injury Cardiomyopathy Shock
**Neurological**	Acute disseminated encephalomyelitis (ADEM) Acute hemorrhagic necrotizing encephalopathy Encephalopathy Generalized myoclonus Gullain-Barré syndrome (acute polyradiculoneuritis) Meningoencephalitis Posterior reversible encephalopathy syndrome (PRES)
**Inflammatory**	Auto-antibody mediated manifestations Exuberant inflammatory response Kawasaki disease Toxic shock syndrome
**Secondary infections**	Bacterial/fungal coinfections

**Table 2:** Original studies regarding the incidence of hyponatremia in COVID-19 patients.

Authors	Reference	Number of Patients	Incidence of Hyponatremia %
Berni, A.; et al.	[13]	29	51.72
Choi, K.W.; et al.	[21]	267	60
Hu, W.; et al.	[31]	1254	9.9
De Carvalho, H.; et al.	[32]	323	31

## Data Availability

Not applicable.
